# Revision by S2-alar-iliac instrumentation reduces caudal screw loosening while improving sacroiliac joint pain—a group comparison study

**DOI:** 10.1007/s10143-020-01377-1

**Published:** 2020-09-10

**Authors:** Sandro M. Krieg, Nico Sollmann, Sebastian Ille, Lucia Albers, Bernhard Meyer

**Affiliations:** 1 Department of Neurosurgery, Klinikum rechts der Isar, Technische Universität München, Ismaninger Str. 22, 81675 Munich, Germany; 2 TUM-Neuroimaging Center, Klinikum rechts der Isar, Technische Universität München, Munich, Germany; 3 Department of Diagnostic and Interventional Neuroradiology, Klinikum rechts der Isar, Technische Universität München, Ismaninger Str. 22, 81675 Munich, Germany

**Keywords:** S2AI, Instrumentation, Sacrum, Degenerative spine, Screw loosening

## Abstract

Lumbosacral instrumentation continues to be challenging due to complex biomechanical force distributions and poor sacral bone quality. Various techniques have therefore been established. The aim of this study was to investigate the outcome of patients treated with S2-alar-iliac (S2AI), S2-alar (S2A), and iliac (I) instrumentation as the most caudal level. Sixty patients underwent one of the 3 techniques between January 2012 and June 2017 (S2AI 18 patients, S2A 20 patients, I 22 patients). Mean age was 70.4 ± 8.5 years. Screw loosening (SL) and sacroiliac joint (SIJ) pain were evaluated during the course at 3-month and maximum follow-up (FU). All patients completed 3-month FU, the mean FU period was 2.5 ± 1.5 years (*p* = 0.38), and a median of 5 segments was operated on (*p* = 0.26), respectively. Bone mineral density (BMD), derived opportunistically from computed tomography (CT), did not significantly differ between the groups (*p* = 0.66), but cages were more frequently implanted in patients of the S2A group (*p* = 0.04). SL of sacral or iliac screws was more common in patients of the S2A and I groups compared with the S2AI group (S2AI 16.7%, S2A 55.0%, I 27.3% of patients; *p* = 0.03). SIJ pain was more often improved in the S2AI group not only after 3 months but also at maximum FU (S2AI 61.1%, S2A 25.0%, I 22.7% of patients showing improvement; *p* = 0.02). Even in shorter or mid-length lumbar or thoracolumbar constructs, S2AI might be considered superior to S2A and I instrumentation due to showing lower incidences of caudal SL and SIJ pain.

## Introduction

Instrumentation of the lumbosacral spine continues to be a challenging area in spine surgery, particularly due to complex local anatomy, unique biomechanical force distributions, and comparatively poor sacral bone quality. Various concepts for construct improvement have therefore been developed. However, there is a high rate of screw loosening (SL), instrumentation failure, pseudarthrosis, and sacroiliac joint (SIJ) pain (up to 83%) [1–3].

The Galveston technique and iliac screws were the first approaches trying to improve caudal instrumentation by extending the construct down to the pelvis [4, 5]. However, although iliac screws were proven to be superior to the Galveston technique in terms of construct strength, iliac screws were shown to cause pain due their prominence, ending in revision surgery in about 22% of cases [5, 6]. Moreover, instrumentation down to the sacrum puts a long cranial cantilever on the SIJ, not only reducing the durability of the construct but also causing a biomechanical overload and therefore severe pain of the SIJ, which was reported in a systematic review to occur in 37 ± 28.48% (range 6–75%) of patients [7].

The search for alternative techniques to this point ended in the description and clinical establishment of the S2-alar-iliac (S2AI) screw trajectory [8]. By crossing 3 cortical bone structures (sacral bone plus SIJ) and allowing for long screw pinching between the compact bone of the pelvis, while also allowing for small incisions due to diverging trajectories and in-line rod bending, this technique offers a variety of advantages over iliac screw placements [9]. Likewise, due to the rigid trans-SIJ trajectory, this technique inhibits any SIJ motion, in contrast to iliac screws and is therefore assumed to better avoid postoperative SIJ pain. On the other hand, some colleagues argue that this rigid SIJ fixation causes reduced but necessary motion inside the SIJ, thus potentially reducing the patients’ quality of life [10]. Moreover, the trajectory is more demanding, thus causing potentially more vascular complications inside the small pelvis or even requiring spinal navigation to ease the approach [11].

**Fig. 1:**
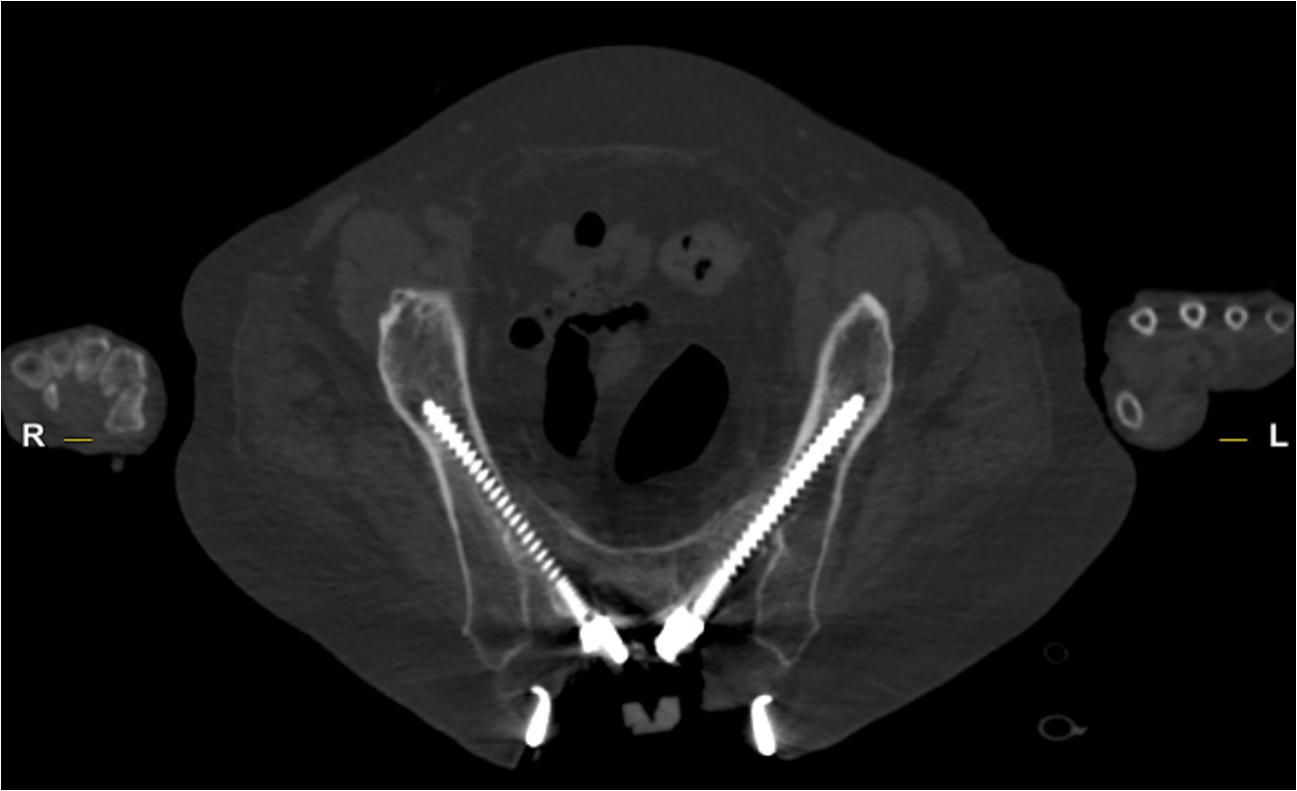
S2-alar-iliac technique. This slide shows the axial computed tomography (CT) slice of bilateral S2-alar-iliac (S2AI) screws. The entry point is between the dorsal S1 and S2 foramens. The screw then crosses the sacroiliac joint into the pelvis above the iliac notch via a trajectory targeting the head of the femur

We therefore hypothesize that instrumentations including S2AI screws are less prone to SL and SIJ pain compared with other established techniques. Thus, the aim of this study was to investigate differences in outcome between patients treated with S2AI, S2-alar (S2A), and iliac (I) instrumentation as the most caudal level.

## Materials and methods

### Study design

We reviewed all medical records and specifically checked the pre- and postoperative examinations including imaging studies, as well as postoperative follow-up (FU). The study enrolled 60 consecutive patients (35 male and 25 female) who underwent instrumentation by one of the 3 techniques between January 2012 and June 2017 as revision surgery after initial instrumentation (S2AI 18 patients [Fig. 1], 50% females, 72.1 ± 7.4 years; S2A 20 patients, 35% females, 69.8 ± 8.6 years; I 22 patients, 41% females, 69.5 ± 9.0 years). Outcome (SL and gluteal pain due to SIJ pain) was compared between the 3 groups considering preoperative, 3-month FU, and maximum FU examinations. Pain was routinely evaluated over the whole observation period during treatment and FU using a 10-point visual analogue scale.

Bone mineral density (BMD) was opportunistically assessed in preoperative imaging by computed tomography (CT) [12]. The 3 groups were comparable regarding the surgical approach and strategy of the treatment, especially in terms of the degree to which their deformities were corrected. The decision to perform one procedure versus another was based on the surgeons’ general preference. No patient criteria were taken into account in making this decision. SL was determined via CT images in all 3 planes by a conference of neurosurgeons and neuroradiologists.

### Ethics

This study was approved by our local ethics committee (registration number: 159/16S) and was conducted in accordance with the Declaration of Helsinki. Informed consent was not required due to the retrospective character of the study and the conditions of our local ethics committee.

### Statistics

All analyses were performed with the statistical software R (version 3.6.1; https://www.r-project.org/). *p* values < 0.05 were considered statistically significant.

General characteristics of the investigated cohort were presented stratified in 3 groups: S2AI, S2A, and I, depending on the surgical technique. Means and standard deviation (SD), median and ranges, or absolute or relative numbers were calculated. Depending on data distribution, analyses of variance (ANOVA), Kruskal-Wallis tests, or Chi-squared tests were used to assess differences in the analyzed parameters between the 3 groups.

The proportions and absolute numbers of patients with SIJ pain according to preoperative assessment, as well as at 3-month and maximum FU, were calculated stratified by group, respectively. Furthermore, proportions and absolute numbers of patients with improvement, deterioration, and unchanged status in SIJ pain at maximum FU compared with the preoperative status were calculated. Differences in these proportions between the 3 groups were assessed using Chi-squared or Fisher’s exact tests.

Potential predictors for improvement in SIJ pain were assessed, again stratified by group. Specifically, mean and SD, median and ranges, or absolute or relative numbers were calculated in patients with improvement and in patients with deterioration or unchanged status regarding SIJ pain. Associations with this status were tested using *t* tests, Mann-Whitney *U* tests, or Chi-squared tests. Improvement at maximum FU compared with the preoperative status was considered. The Benjamini-Hochberg procedure was used for correction of multiple testing regarding these prediction analyses, assuming a false discovery rate of 25%.

## Results

### General results and group differences

Participants’ mean age was 70.4 ± 8.5 years. The general demographics of all 3 groups are outlined in Table 1. Overall, the 3 groups were highly comparable and showed no statistically significant differences regarding baseline characteristics at the preoperative state. All patients received a 2-rod titanium construct.

**Table 1:** General characteristics

		S2AI (*n* = 18)	S2A (*n* = 20)	I (*n* = 22)	*p*
Age (in years; mean (SD))		72.1 (7.4)	69.8 (8.6)	69.5 (9.0)	0.364
Sex (% of patients (*N*))	Female	50.0 (9)	35.0 (7)	40.9 (9)	0.646
Osteopenia / osteoporosis (% of patients (*N*))	None	0.0 (0)	10.5 (2)	11.1 (2)	0.607
	Osteopenia	29.4 (5)	36.8 (7)	22.2 (4)	
	Osteoporosis	70.6 (12)	52.6 (10)	66.7 (12)	
Spondylolisthesis (% of patients (*N*))		27.8 (5)	20.0 (4)	36.4 (8)	0.551
Previous surgery					
No. of decompressed vertebrae (median (range))		3.0 (0–6)	2.0 (0–4)	2.5 (0–5)	0.300
No. of fused segments (median (range))		3.0 (0–7)	3.0 (0–6)	3.0 (0–8)	0.664
No. of screws one side (median (range))		4.0 (0–6)	4.0 (0–7)	3.5 (0–9)	0.865
Cement augmentation (% of patients (*N*))		6.3 (1)	12.5 (2)	25.0 (5)	0.311
No. of cages (median (range))		0.5 (0–4)	0.5 (0–3)	1.0 (0–8)	0.731
Vertebral body replacement (% of patients (*N*))		6.3 (1)	6.3 (1)	20.0 (4)	0.351
Anterior fusion (% of patients (*N*))		12.5 (2)	0.0 (0)	10.0 (2)	0.537
Surgery					
Time since last spine surgery (in years; mean (SD))		2.0 (2.7)	1.7 (2.7)	1.4 (1.1)	0.458
Screw length (in mm; mean (SD))		82.8 (9.0)	49.3 (7.5)	97.7 (13.1)	*0.014*
No. of decompressed vertebrae new (median (range))		0.5 (0–5)	1.0 (0–7)	0.0 (0–6)	*0.039*
No. of fused segments overall (median (range))		5.0 (2–9)	4.5 (1–9)	3.0 (2–11)	0.258
No. of screws one side overall (median (range))		6.0 (3–11)	6.5 (3–11)	5.0 (3–13)	0.294
Cement augmentation new (% of patients (*N*))		27.8 (5)	35.0 (7)	22.7 (5)	0.675
No. of levels with newly implanted cages (median (range))		0.0 (0–4)	1.5 (0–5)	0.0 (0–3)	*0.039*
Newly implanted vertebral body replacement (% of patients (*N*))		5.6 (1)	15.0 (3)	4.6 (1)	0.507
Additional anterior fusion (% of patients (*N*))		0.0 (0)	5.0 (1)	4.6 (1)	1.000
Intraoperative blood loss (in ml; mean (SD))		1911.0 (2523.0)	1120.0 (593.3)	1545.0 (1436.0)	0.899
Duration of surgery (in min; mean (SD))		289.3 (98.3)	247.4 (73.6)	248.0 (88.3)	0.245

General characteristics of the investigated cohort divided into three groups: S2-alar-iliac (S2AI), S2-alar (S2A), and iliac (I) instrumentation. Furthermore, information on the preoperative status regarding previous surgery as well as info on characteristics of the present surgery are given. Italicized *p* values indicate statistical significance (*p* < 0.05)

All patients completed 3-month FU; maximum FU times were 2.3 ± 0.9 (S2AI), 3.0 ± 1.7 (S2A), and 2.4 ± 1.6 (I) years (*p* = 0.38). A median of 4.5 to 5 segments (S2AI, S2A) and 3 segments (I) was operated on (*p* = 0.26), extending to S2 or the os ilium, respectively. BMD as assessed opportunistically by CT did not significantly differ between the groups (*p* = 0.66). Cages were more frequently implanted in patients of the S2A group (*p* = 0.04).

### Caudal screw loosening

SL of sacral or iliac screws was more common in patients of the S2A and I groups when compared with the S2AI group (S2AI 16.7%, S2A 55.0%, I 27.3% of patients; *p* = 0.03).

### Improvement in sacroiliac joint pain

Gluteal pain as a clinical sign for SIJ pain was more often improved in the S2AI group not only after 3 months but also at maximum FU (S2AI 61.1%, S2A 25.0%, I 22.7% of patients showing improvement; *p* = 0.02; Table 2). While the S2A group already had a low rate of preoperative SIJ pain, the pain was comparable in the S2AI and I groups but significantly better in the S2AI group at 3-month and maximum FU. While 61.1% of patients in the S2AI group improved from their preoperatively existing SIJ pain, only 25.0% in the S2A and 18.2% in the I group improved at 3-month FU.

**Table 2:** Improvement in sacroiliac joint (SIJ) pain

		S2AI (*n* = 18)	S2A (*n* = 20)	I (*n* = 22)	*p*
		% (*N*)			
SIJ pain—preoperative (% of patients (*N*))		61.1 (11)	30.0 (6)	63.6 (14)	0.059
SIJ pain—3-month follow-up (% of patients (*N*))		27.8 (5)	10.0 (2)	40.9 (9)	0.078
SIJ pain—maximum follow-up (% of patients (*N*))		11.1 (2)	10.0 (2)	40.9 (9)	*0.030*
SIJ pain—3-month follow-up compared with preoperative status (% of patients (*N*))	Worse	5.6 (1)	0.0 (0)	0.0 (0)	*0.007*
	Better	61.1 (11)	25.0 (5)	18.2 (4)	
	Unchanged	33.3 (6)	75.0 (15)	81.8 (18)	
SIJ pain—maximum follow-up compared with preoperative status	Worse	5.6 (1)	0.0 (0)	0.0 (0)	*0.015*
	Better	61.1 (11)	25.0 (5)	22.7 (5)	
	Unchanged	33.3 (6)	75.0 (15)	77.3 (17)	

Overview on SIJ pain during the clinical course considering the preoperative status as well as assessments at 3-month and maximum follow-up (FU). Italicized *p* values indicate statistical significance (*p* < 0.05)

Specifically, predictors of SIJ pain at maximum FU were the number of fused segments and the number of screws per side from previous surgery among the S2AI group (Table 3), but these predictors did not survive correction for multiple comparisons. However, patients with SIJ pain at FU also required revision surgery significantly more often, in most cases due to and as a sign of SL.

**Table 3:** Sacroiliac joint (SIJ) pain at maximum follow-up (FU)

		S2AI (*n* = 18)			S2A (*n* = 20)			I (*n* = 22)		
		Unchanged or worse (*N* = 7)	Improved (*N* = 11)	*p*	Unchanged or worse (*N* = 15)	Improved (*N* = 5)	*p*	Unchanged or worse (*N* = 17)	Improved (*N* = 5)	*p*
Age (in years; mean (SD))		72.1 (6.7)	72.1 (8.4)	0.992	69.7 (9.11)	70.1 (8.9)	0.931	70.7 (10.0)	65.6 (4.5)	0.125
Sex (% of patients (*N*))	Female	71.4 (5)	36.4 (4)	0.335	60.0 (9)	80.0 (4)	0.613	58.8 (10)	60.0 (3)	1.000
Osteopenia/osteoporosis (% of patients (*N*))	None	0.0 (0)	0.0 (0)	0.593	14.3 (2)	0.0 (0)	0.061	6.7 (1)	33.3 (1)	0.407
	Osteopenia	42.9 (3)	20.0 (2)		50.0 (7)	0.0 (0)		26.7 (4)	0.0 (0)	
	Osteoporosis	57.1 (4)	80.0 (8)		35.7 (5)	100.0 (5)		66.7 (10)	66.7 (2)	
Spondylolisthesis (% of patients (*N*))		42.9 (3)	18.2 (2)	0.326	20.0 (3)	20.0 (1)	1.000	29.4 (5)	60.0 (3)	0.309
Previous surgery										
No. of decompressed vertebrae (median (range))		3.0 (0–4)	3.0 (0–6)	1.000	2.0 (0–4)	2.5 (0–3)	1.000	2.0 (0–5)	3.0 (2–4)	0.392
No. of fused segments (median (range))		2.0 (0–4)	4.0 (0–7)	0.035*	2.5 (0–5)	3.0 (0–6)	0.530	2.0 (0–8)	4.0 (2–6)	0.132
No. of screws one side (median (range))		3.0 (0–4)	5.0 (0–6)	0.020*	4.0 (2–5)	4.0 (0–7)	0.935	3.0 (0–9)	4.0 (3–6)	0.127
Cement augmentation (% of patients (*N*))		14.3 (1)	0.0 (0)	0.438	8.3 (1)	25.0 (1)	0.450	20.0 (3)	40.0 (2)	0.560
No. of cages (median (range))		0.0 (0–2)	1.0 (0–4)	0.394	0.5 (0–3)	1.0 (0–3)	0.948	1.0 (0–8)	2.0 (0–4)	0.114
Vertebral body replacement (% of patients (*N*))		0.0 (0)	11.1 (1)	1.000	8.3 (1)	0.0 (0)	1.000	13.3 (2)	40.0 (2)	0.249
Surgery										
Time since last spine surgery (in years; mean (SD))		1.7 (2.6)	2.2 (2.9)	0.725	2.1 (3)	0.6 (0.4)	0.120	1.2 (0.8)	2.1 (1.7)	0.292
Screw length (in mm; mean (SD))		80.0 (8.2)	84.6 (9.3)	0.295	48.3 (8.2)	52.0 (4.5)	0.229	96.5 (14.1)	102.0 (8.4)	0.298
No. of decompressed vertebrae new (median (range))		0.0 (0–2)	1.0 (0–5)	0.697	2.0 (0–7)	1.0 (0–2)	0.301	0.0 (0–6)	0.0 (0–0)	0.146
No. of fused segments overall (median (range))		5.0 (2–9)	5.0 (2–8)	0.520	4.0 (1–9)	5.0 (2–8)	1.000	3.0 (2–11)	6.0 (2–8)	0.375
No. of screws one side overall (median (range))		5.0 (3–11)	6.0 (3–9)	0.583	6.0 (3–11)	7.0 (4–10)	0.755	5.0 (3–13)	7.0 (5–10)	0.096
Cement augmentation new (% of patients (*N*))		0.0 (0)	45.5 (5)	0.101	40.0 (6)	20.0 (1)	0.613	29.4 (5)	0.0 (0)	0.290
No. of levels with newly implanted cages (median (range))		0.0 (0–3)	0.0 (0–4)	0.909	1.0 (0–5)	2.0 (0–4)	0.853	0.0 (0–3)	0.0 (0–1)	0.831
Newly implanted vertebral body replacement (% of patients (*N*))		14.3 (1)	0.0 (0)	0.389	20.0 (3)	0.0 (0)	0.539	5.9 (1)	0.0 (0)	1.000

Potential predictors for an improvement in SIJ pain are shown in this table, differentiating between patients with improvement and patients with deterioration or unchanged status regarding SIJ pain. Italicized *p* values indicate statistical significance (*p* < 0.05); *p* values with asterisks did not survive correction for multiple comparisons using the Benjamini-Hochberg procedure with a false discovery rate of 25%

### Complications

There were no perioperative screw-related vascular or visceral surgical complications due to sacral or iliac screws; however, there was one screw-related aortic dissection due to an L2 screw in the I group. In the S2AI group, cerebrospinal fluid (CSF) leakage due to surgery was observed in 3 patients (16.7%), whereas hematoma occurred in 1 patient (5.6%). Furthermore, 3 patients (15.0%) of the S2A group showed CSF leakage, and 2 (10.0%) showed hematoma, whereas another 2 patients (10.0%) showed screw dislocation with cement leakage in 1 case. Among patients of the I group, 1 patient (4.5%) showed screw dislocation.

Regarding postoperative medical complications, urinary tract infection was most common (S2AI 4 patients, S2A 2 patients), followed by deep vein thrombosis (S2AI 2 patients, S2A 1 patient, I 2 patients), partially with related pulmonary artery embolism (S2AI 1 patient, I 2 patients). Two patients of the S2A group developed pneumonia, 1 patient of the S2AI group had a postoperative non-ST-elevation myocardial infarction, and 1 patient of the I group had postoperative endocarditis.

## Discussion

Our main findings were that S2AI showed superiority compared with S2A and I techniques in terms of reduced caudal SL and a lower rate of SIJ pain. Additionally, none of the differences between groups significantly promoted these effects.

Considering the results, any of the 3 techniques is better than using S1 instrumentation as the caudal part at all [3]. While spinopelvic fixation is nowadays achieved via a range of approaches, iliac and S2AI screws are presently the most commonly used ones. Caudal instrumentation down to the pelvic ring offers reinforced biomechanical strength, especially if long fusions extend to the sacrum or in cases of sacrectomy, osteoporosis, and deformity surgery necessitating osteotomies or general revisions [13]. Clinically, additional iliac screws have been shown to be superior in neuromuscular spinal deformities and overall pediatric patients [14]. The same is true in adult revision cases after failed lumbosacropelvic fixation. Even in those cases, lumbosacral fusion could be promoted [15].

Although literature reports a considerably high rate of lumbosacral non-fusion of up to 83%, the rate was considerably lower in our series, despite the analyzed groups and despite all cases being revision cases [1, 2, 16–18]. Although iliac screws became standard over the last decade in many centers due to superior construct endurance, screw prominence can induce pain, sitting difficulty, and can even require screw removal [1, 19, 20]. However, this was not the case in our iliac screw cohort. Despite new technical reports on reducing iliac screw prominence, S2AI screws do not harbor this issue at all [21]. Typically, and as our series does, such surgeries harbor not only an increased risk of perioperative surgical but also medical complications [22], despite the outcome usually being affected [23].

The current best level of evidence originates from a meta-analysis of 5 retrospective studies reporting a significantly lower rate of revision surgery, wound infection, and screw-related pain for S2AI versus iliac screws [22]. However, this study reported heterogeneous data of different centers with therefore even reported selection bias. Furthermore, although our study, as does the reported one, also reflects level III evidence, it not only reports on homogeneous single-center data but also investigates the potential of such instrumentation to treat SIJ pain originating from overstressed SIJ due to long cranial cantilever transmitted by the sacral instrumentation, which is a sparsely investigated issue. While another study showed that S2AI screws cause less postoperative SIJ pain compared with S1, S2, or L5 screws as the caudal end of the construct, our study actually shows that in this matter, S2AI screws are even superior to iliac screws [24]. With these results, our study is the first to prove not only that S2AI screws improve already existing SIJ pain, but also the superiority of this approach to iliac screws. Although both techniques bridge the SIJ, there seems to be some remaining minor but still sufficient movement within the SIJ in case that iliac screws are used. S2AI screws, in contrast, go directly through the SIJ and therefore cease even minor movement within the SIJ. This not only is relevant surgically considering SIJ pain as a disabling sequelae of sacral instrumentation but also shows us, impressively, how little movement within the SIJ is able to elicit this pain and how large the forces onto the SIJ after instrumentation down to the sacrum need to be. This is relevant especially when considering trans-SIJ plating as a sufficient treatment of SIJ pain.

In previous studies, such as the already mentioned meta-analysis, there was a difference in indications for spinopelvic instrumentation and in the rate of patients receiving anterior cage support [22], which was not the case in our series. Furthermore, the lower rate of SL of the S2AI screws compared with sacral and iliac screws is well in accordance with previous data, which seems mostly due to omission of connectors as a potential source of failure and the stronger cortical purchase by crossing 3 cortical bone structures. Crossing 3 cortical bone structures seems to be the main issue defining the persistence of the S2AI screws. A cadaveric study proved that 65-mm S2AI screws were as strong as 80-mm S2AI and 90-mm iliac screws, showing that the tricortical purchase and not the overall length seems decisive [25]. Considering additional anterior column support via the anterior, oblique, and lateral of posterior cages is regarded as essential to promote fusion and relieve stress from posterior elements [13, 26]. Despite our cohort reporting a direct comparison of 3 very homogeneous groups of one center, mean FU was long enough to be within the commonly reported time prone to SL. Nonetheless, our cohort does not report standardized questionnaires and can therefore only report the subjectively experienced gluteal pain plus the objectively detected SL.

## Conclusion

In conclusion, even in shorter or mid-length lumbar or thoracolumbar constructs, S2AI might be considered superior to S2A and I instrumentation due to lower incidences of caudal SL and SIJ pain. However, the superiority not only is in the outcome, but the surgical technique also provides some advantages by allowing for small incisions due to diverging trajectories, less dissection, and in-line rod bending without the need for additional connectors. Because the trajectory is more demanding, thus potentially causing more anterior complications, we recommend spinal navigation to ease the approach, as done in all cases of this study. Although our data might be partially biased, the results and clinical experience are clear. We therefore advocate for the S2AI technique as the caudal end of longer instrumentations. Future prospective studies enrolling larger series should be conducted to confirm these initial results.

## Data Availability

The data that support the findings of this study are available from the corresponding author, upon reasonable request.
